# Brachyury: A sensitive marker, but not a prognostic factor, for skull base chordomas

**DOI:** 10.3892/mmr.2015.3976

**Published:** 2015-06-22

**Authors:** KE WANG, KAIBING TIAN, LIANG WANG, ZHEN WU, CONG REN, SHUYU HAO, JIE FENG, JUNHUA LI, HONG WAN, GUIJUN JIA, LIWEI ZHANG, JUNTING ZHANG

**Affiliations:** 1 Department of Neurosurgery, Skull Base and Brainstem Tumor Division, Beijing Tian Tan Hospital, Capital Medical University, China National Clinical Research Center for Neurological Diseases, Beijing 100050, P.R. China; 2 Beijing Neurosurgery Institute, Capital Medical University, Beijing 100050, P.R. China

**Keywords:** skull base chordoma, prognosis, Brachyury, radical surgery

## Abstract

Patients with skull base chordomas have a poor prognosis, and the role of the protein expression of brachyury in chordomas remains to be fully elucidated. The present study used immunohistochemistry to analyze 57 cases of skull base chordoma, and analyzed the clinical data of the patients. The results demonstrated that the protein expression of brachyury was negative in 8.8% (5/57) of the cases. The weak/positive, positive and strong/positive rates were 5.3% (3/5 7), 21.1% (12/57) and 64.9% (37/57), respectively. The association between the expression of brachyury and recurrence was not statistically significant. Kaplan-Meier analysis revealed that the degree of surgery, rather than the expression of brachyury, was associated with tumor recurrence (P=0.001). In conclusion, the results of the present study demonstrated that the expression of Brachyury offers a sensitive marker, but not a risk factor, for skull base chordomas, and radical surgery is recommended.

## Introduction

Chordomas are rare, low-grade malignant tumors, which are derived from the notochord (1,2). The prognosis of these types of tumor is poor as a result of its slow growth and infiltrating characteristics (3). Surgery, combined with adjuvant radiotherapy, is the treatment of preference for treating skull base chordomas (3–5). A systematic review revealed that the 5-year progression-free survival and 5-year overall survival (OS) rates of patients with chondromas were 50.8 and 78.4%, respectively (6). Patients who underwent partial surgery were observed to suffered higher rates of recurrence and shortened OS, compared with those who underwent complete surgery (6).

Brachyury is among the most common protein observed during analyses of chordomas. Brachyury is a protein encrypted by the T gene, which is located at 6q27 and is a relatively well-conserved gene (7). The brachyury gene is associated with chordomas (8), particularly with familial chordomas (9). Previous studies using single nucleotide polymorphism (SNP) methods revealed that the brachyury Gly177Asp SNP (rs2305089 SNP) was associated with chordomas in European populations (10), but not in the Han Chinese population (11). The duplication or gain of the T locus is a common phenomenon in chordomas, with protein expression of brachyury in sporadic and familial chordomas, as well as the primary notochord, but no brachyury protein expression was observed in the nucleus pulposus cell (8,9). The silencing of the gene expression of brachyury in the UCH-1 cell line results in a decrease in cell proliferation (8).

However, not all chordomas express the brachyury protein. Previous data have revealed that the brachyury protein is positively expressed in 75.64–100% of chordomas (7,12–13), and is not a prognostic factor in spinal and sacral chordomas (13). Clinical data of the association between prognosis and the protein expression of brachyury, particularly in skull-based chordomas, is limited. The present study aimed to analyze the association of the brachyury expression and the prognosis of skull-based chordomas.

## Patients and methods

### Patients

A total of 57 skull base chordoma samples were obtained from patients between May 2008 and May 2013 in the Department of Neurosurgery, Beijing Tian Tan Hospital, Capital Medical University (Beijing, China). All cases were confirmed pathologically using traditional hematoxylin and eosin (HE) staining and cytokeratin, S-100, vimentin and epithelial membrane antigen staining. This procedure was performed according to the guidelines of the Department of Neuro-Pathology, Beijing Neurosurgical Institute (Beijing, China). All clinical data (Table I), and magnetic resonance (MR) images and computed tomography (CT) were retrospectively examined in detail. The patients were followed-up with post-surgical office visits and telephone interviews for those who were unable to attend the office. All data were compiled from the hospital and office records, imaging, and records provided by the patients themselves. The present study was performed according to the principles of the Declaration of Helsinki and approved by the Ethical Committee of Beijing Tian Tan Hospital (Beijing, China). All patients provided written informed consent and, for patients <15-years, written consent of the parents, with the fingerprints of the children were provided. The Ethical Committee of Beijing Tian Tan Hospital approved the consent procedure.

### Methods

The chordoma specimens were collected immediately following surgical resection without electrocoagulation. For storage, 10% neutral formalin solution (Beijing Yili Fine Chemical Co., Ltd., Beijing, China) was used. All specimens were subsequently embedded in paraffin within the first week. HE staining was performed prior to brachyury protein staining on 3 mm slices of the specimens. The rabbit polyclonal antibody for brachyury H-210 (1:500; Santa Cruz Biotechnology, Inc., Santa Cruz, CA, USA), which is an antibody raised against amino acids 226–435 of the human brachyury gene was used for immunohistochemical staining. The streptavidin-peroxidase two-step method was performed under the direction of the HRP-DAB Substrate Chromogenic Reagent kit (PA109; Tiangen Biotech (Beijing) Co., Ltd, Beijing, China).

**Figure 1:**
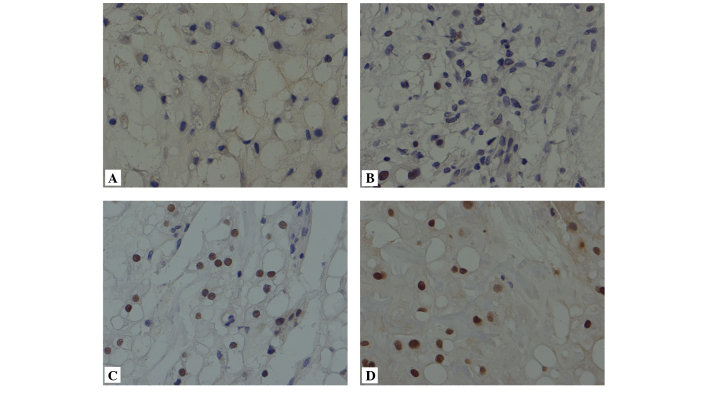
Representative images of immunohistochemical staining to observe the expression of brachyury in skull base chordoma tissues. Images represent (A) negative cells, lacking nuclear staining of tumor cells; (B) 1+ staining, in which ≤30% nuclear staining of tumor cells was observed; (C) 2+ staining, in which 31–60% positive nuclear staining was observed and (D) 3+ staining, in which between 61 and 100% nuclear staining was observed (magnification, x400).

Brachyury-positive samples were defined as those exhibiting nuclear staining, and the brachyury staining patterns were categorized into the following four groups, as previously described (13): 0, no nuclear staining; 1+, ≤30% staining of tumor cells; 2+, 31–60% staining of tumor cells; 3+, 61–100% staining of tumor cells, as shown in Fig. 1. The percentage of cells exhibiting positive nuclear staining for brachyury was calculated as the average number of cells in five fields of view using a high power lens (magnification, x400; Axio Imager A2, Zeiss, Jena, Germany). Three independent pathologists from the Beijing Neurosurgery Institute categorized the brachyury staining in a blinded manner and a consensus was reached in all cases.

The surgical degree was confirmed by the senior surgeons and postoperative MR and CT images as follows: i) gross total resection: no residual tumor detected on the postoperative MR and CT images, as well as during the surgeon's observation under the microscope during surgery; ii) subtotal resection: residual tumor at <10% of the original volume; and iii) partial resection: residual tumor at >10% of the original volume. Gross total and subtotal removal were defined as radical resection. Tumor recurrence was defined as any newly identified enhancement following gross total resection or any increase in the tumor volume following subtotal resection.

### Statistical analysis

The clinical data and follow-up data were collected using Epidata 3.02 database software (EpiData Association, Odense, Denmark). The groups with different expression levels of brachyury and other clinical risk factors were compared. Kaplan-Meier and COX survival analyses, and χ^2^ tests were performed using SPSS 12.0 software (SPSS, Inc., Chicago, IL, USA). P<0.01 was considered to indicate a statistically significant difference.

## Results

The 57 patients were all pathologically confirmed to have skull base chordomas. Of these patients, two were confirmed to exhibit chondroid chordoma and the remaining patients exhibited classic chordomas. The male to female ratio was 1:1.03 (28:29) and the mean age was 35.7±13.0 years (range, 12–61 years). Of the 57 cases, 11 were cases of recurrent skull base chordoma, whereas 46 of the cases were present in untreated patients. The tumor volumes ranged between 5 and 168 ml (mean, 35±30 ml), and surgery was performed by senior neurosurgeons, predominantly via the lateral approach. Overall, 20 patients underwent total tumor resection, 29 patients underwent subtotal resection and eight patients underwent partial tumor resection. Several complications were observed during the post-operative period. A number of patients required tracheotomies and received antibiotic treatment for intracranial (15 patients) or pulmonary infections (2 patients), and one patient died due to untreatable pulmonary infection following complete tumor resection and tracheotomy.

During the average follow-up time of 19.6 months (range, 4–66 months), 10 patients succumbed to illness, of which one patient exhibited pulmonary metastasis and undergone surgery three times prior to admission to the Department of Neurosurgery, Beijing Tian Tan Hospital. Among the 11 cases of recurrent skull base chordoma, five patients succumbed to illness, eight of which (8/11, 72.7%) were due to tumor recurrence or metastases. There were 18 cases of recurrence following surgical treatment in the Department of Neurosurgery, and the mean recurrence period was 9.9 months (range, 1–36 months).

All 57 cases were stained with brachyury, of which two chondroid skull base chordomas exhibited staining of 2+ and 3. The results of the staining demonstrated that five cases were negative for staining (8.8%), three cases exhibited weak positive staining (5.3%), 12 cases exhibited 2+ positive staining (21.1%) and 37 cases exhibited 3+ strong positive staining (64.9%), as shown in Fig. 1. The different protein expression levels of brachyury were not associated with the tumor volume, degree of resection, previous history, post-operative recurrence or follow-up disease state (Table I).

**Figure 2:**
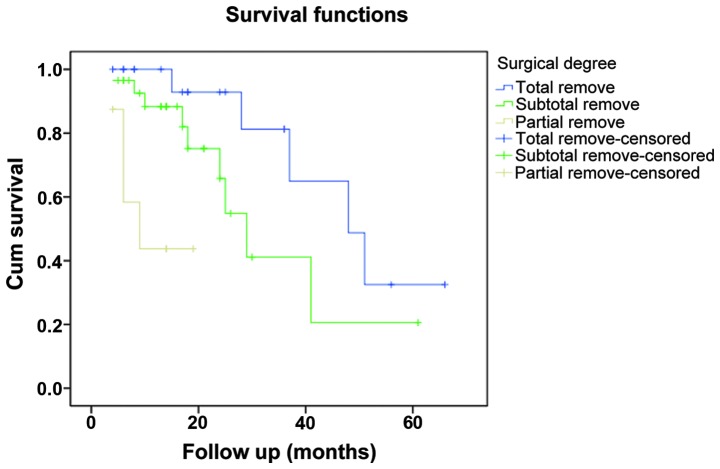
Kaplan-Meier analysis of the association between the three surgical degrees and recurrence (P=0.001). Cum, cumulative.

The age of the patients (<20, 20–45 or >45-years), expression levels of brachyury, gender, disease state (primary or recurrent), tumor volume (<20, 20–60 or >60 ml), degree of surgical removal (total, subtotal or partial) and presence or absence of post-operative radiotherapy were analyzed using the COX multiple survival test (data not shown). The results revealed that the degree of surgery was the only risk factor that was associated with recurrence and survival rate (P=0.003 and P=0.022, respectively). The Kaplan-Meier survival analysis confirmed that the surgical degree was associated with recurrnce (P = 0.001; Fig. 2).

**Figure 3:**
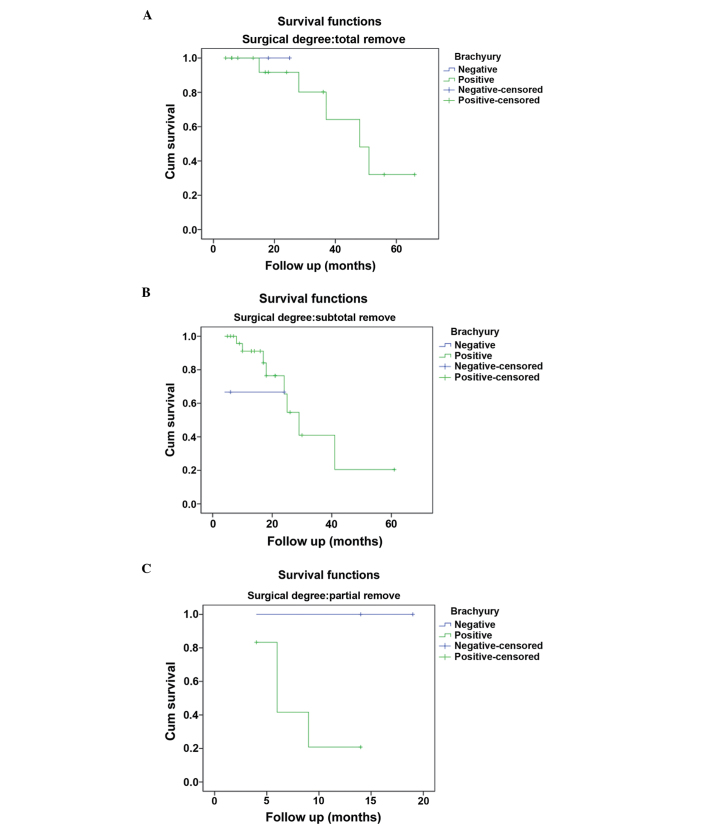
Kaplan-Meier analysis of the expression levels of brachyury and recurrence in the (A) total removal (P=0.683), (B) subtotal removal (P=0.418) and (C) partial removal (P=0.112) groups. Cum, cumulative.

With different degrees of surgical removal, the association between the expression of brachyury and recurrence was analyzed using Kaplan-Meier analysis. Since the number of cases with negative expression of brachyury was limited, the expression level of Brachyury was combined into two groups for analysis: Group 1, negative and weak positive expression; group 2, positive and the strong positive expression). However, the results demonstrated no association between the expression of brachyury and recurrence in any of the three degrees of surgical removal (Fig. 3; P>0.05).

The two cases of chondroid skull base chordomas were primary cases with a follow-up of 13 months, and they exhibited were high on the follow-up Karnofsky performance scale, without recurrence (90/100).

## Discussion

Skull base chordomas are derived from remnants of the notochord and are often observed between the third and fifth decade of life, without notable gender predominance (1–2). Skull base chordomas have a poor prognosis due to the invasiveness to vital structures, thereby complicating surgical treatment, since total removal may have a high risk of complications and result in a poor quality of life (14). However, total removal has been advocated in previous reports due to concern regarding tumor recurrence (4,6). In a previous study of 132 craniocervical area chordomas, total or radical (subtotal) surgery was performed, resulting in 5- and 10-year survival rates of 55 and 36%, respectively (14). In the present study, radical surgery was performed, particularly in patients with primary disease (4). The relatively short follow-up periods in the present study demonstrated that the degree of surgery was a risk factor for recurrence, and that patients with total tumor removal had a longer tumor progression-free period, compared with those who received subtotal or partial removal.

The present study demonstrated that the brachyury protein was expressed in 91.2% of skull base chordomas. This was higher, compared with the expression level reported by Zhang *et al* (13). in which the protein expression of brachyury in spine and sacrum chordomas, was 75.64% (59/78), based on tissue microarray staining. Jambhekar *et al* (12) reported in their investigation of 51 cases, that brachyury protein was expressed in 46 cases (90.2%), including those with chondroid components. The results of the present study were similar to those of Jambhekar *et al* (12), with high levels of brachyury-positive expression and positive staining for bracyhury obsetved in two chondroid chordomas. A review of the literature analyzing the expression rate of brachyury in axial chordomas (Table II), revealed that the expression rate of brachyury was 87.0% (75.64–100%), demonstrating that brachyury was relatively sensitive for diagnosis, including for tumors located in the extra-axial spaces (15).

Brachyury acts as a key factor for the epithelial to mesenchymal transition of human carcinoma cell lines and promotes the metastatic dissemination of human tumor xenografts *in vivo* (16). The protein expression level of brachyury is positively correlated with the resistance of malignant cells to various chemotherapeutic and irradiation treatments (7,8,12). It has been reported that the protein expression of brachyury is associated with the prognoses of primary lung carcinoma (17) and colorectal cancer (18). The brachyury protein has also been associated with the prognosis of patients with skull base chordomas (19), however, the results reported by Zhang *et al* (13) revealed that the protein expression of brachyury is not associated with the prognosis of spinal or sacral chordomas. In the present study, the majority of the skull base chordomas were positive for brachyury protein, indicating that it was the degree of surgery, rather than the expression of brachyury, which was associated with tumor recurrence.

In the present study, not all the cases were brachyury-positive. In addition, among the patients with a brachyury-positive tumor, brachyury-negative tumor cells were present, as shown in Fig. 1. Shen *et al* (20) reported that chordoma cells and benign notochord cells can be detected in the same specimen, which may explain the difference in the expression of brachyury in the same lesion as benign notochord cells are negative for brachyury staining. In addition, Kitamura *et al* (19) revealed that the brachyury-negative chordomas were different compared with the brachyury-positive chordomas. Of the three types of chordomas, which consist of the classic, chondroid and dedifferentiated types (1–4), the chondroid type has been demonstrated to be brachyury-positive, which was observed in the present study in the two chondroid chordomas (7,12–13). Classic chordomas are predominantly positive for Brachyury, however, whether the dedifferentiated chordomas are positive for brachyury remains to be elucidated, partly due to its rarity. The reason for the expression of brachyury in chordomas, which is suggested to be due to the copy number gain of the T gene (gain of the 6q gene) (8,19,21), remains to be fully elucidated. However, use of the brachyury protein as a sensitive marker for chordomas may be an appropriate biomarker for future molecular therapeutic targeting (19).

In conclusion, the present study, which investigated 57 cases of skull base chordoma, demonstrated that the expression of brachyury can be used as a sensitive marker, rather than as a prognostic factor. However, the degree of surgery is a prognostic factor for skull base chordomas, and radical surgery is advocated. Further investigations are required to determine the regulation of the expression of brachyury.

## Figures and Tables

**Table I:** Association between the clinical data and the differential expression levels of Brachyury protein.

Parameter	n (%)	Degree of staining				P-value
		0	1+	2+	3+	
Age (years)						
<20	14 (24.6)	1	1	0	12	0.068
20–45	29 (50.9)	2	1	9	17	
>45	14 (24.6)	2	1	3	8	
Gender						
Male	28 (49.1)	3	0	6	19	0.935
Female	29 (50.9)	2	3	6	18	
Primary disease status						
Primary	46 (80.7)	4	3	7	25	0.559
Recurrent	11 (19.3)	1	0	5	12	
Tumor volume (ml)						
<20	26 (45.6)	1	1	5	19	0.794
20–60	21 (36.8)	0	2	5	14	
>60	10 (17.5)	4	0	2	4	
Surgical removal						
Total	20 (35.1)	2	1	3	14	0.277
Subtotal	29 (50.9)	2	1	6	20	
Partial	8 (14.0)	1	1	3	3	
Adjuvant radiation						
Yes	16 (28.1)	0	0	5	11	0.444
No	41 (71.9)	5	3	7	26	
Follow up						
No recurrence	22 (38.6)	2	0	2	18	0.143
Recurrence	25 (43.9)	2	2	7	14	
Mortality	10 (17.5)	1	1	3	5	

Recurrence refers to the recurrence following surgical removal at follow-up. Degree of staining: 0, no nuclear staining; 1+, ≤30% staining; 2+, 31–60% staining; 3+, 61–100% staining.

**Table II:** Different expression levels of Brachyury, previously reported.

Year	Author	Brachyury positive (positive/total; n)	Tumor site	Positive expression rate (%)
Axial				
2013	Zhang *et al* (13)	59/78	Spine and sacrum	75.64
2010	Jambhekar *et al* (12)	46/51	Skull base, spine and sacrum	90.2
2013	Kitamura *et al* (19)	30/37	Skull base	81.1
2006	Vujovic *et al* (7)	53/53	Not stated	100
Present	Wang *et al*	52/57	Skull base	91.2
Total		240/276		87.0
Extra-axial				
2008	Tirabosco *et al* (15)	10/12	Extra-axial	83.3
